# Effectiveness of acceptance and commitment therapy on weight, eating behaviours and psychological outcomes: a systematic review and meta-analysis

**DOI:** 10.1007/s40519-023-01535-6

**Published:** 2023-02-10

**Authors:** Han Shi Jocelyn Chew, Samuel Chng, Nagadarshini Nicole Rajasegaran, Khun Hean Choy, Yuen Yu Chong

**Affiliations:** 1 Alice Lee Centre for Nursing Studies, Yong Loo Lin School of Medicine, National University of Singapore, Singapore, Singapore; 2 Lee Kuan Yew Centre for Innovative Cities, Singapore University of Technology and Design, Singapore, Singapore; 3 The Nethersole School of Nursing, Faculty of Medicine, The Chinese University of Hong Kong, Hong Kong, China

**Keywords:** Acceptance commitment therapy, ACT, Weight, Obesity, Behaviour change, Eating, Psychological flexibility

## Abstract

**Purpose:**

To examine the effectiveness of ACT on weight (body mass index and body mass), eating behaviours (binge eating, emotional eating, external eating and restraint eating), and psychological outcomes (quality of life [QoL], depression, psychological flexibility, and weight stigma) among adults with overweight and obesity.

**Methods:**

Seven electronic databases (CINAHL, EMBASE, PubMed, PsycInfo Scopus, The Cochrane Library, and Web of Science) were searched from inception through 17 June 2022. 13 studies and 48 unique effect sizes were analyzed using random-effects models. Pooled effect estimates were calculated using weighted mean differences (WMD) and standardized mean differences expressed in Hedges’ *g* (*g*). Heterogeneity was assessed using Q-statistics and interpreted using *I*^2^.

**Results:**

ACT was found to be effective in improving weight loss in terms of BMI (*k* = 6, WMD = − .50, 95% CI = − .90; − .11, *t* = − 3.25, *p* = .20, *I*^2^ = .0%), psychological flexibility and weight-related stigma. However, non-significant changes were found for body mass (*k* = 4, WMD = − 0.33 95% CI = − 1.53; 0.87, *t* = − .88, *p* = .44, *I*^2^ = .0%), binge eating (*k* = 4, *g* = − .34, 95% CI = − 1.31; 0.62, *t* = − 1.13, *p* = .34, *I*^2^ = 71.1%), emotional eating (*k* = 6, *g* = − .20, 95% CI = − 0.54; 0.15, *t *= − 1.47, *p *= .20, *I*^2^ = 45.0%), external eating (*k* = 5, *g* = − .40, 95% CI = 0.96; 0.16, *t* = − 1.99, *p* = .12, *I*^2^ = 81.8%), restraint eating (*k* = 3, *g* = .22 95% CI = − 0.57; 1.01, *t* = 1.19, *p* = .36, *I*^2^ = 69.1%), QoL (*k* = 3, *g* = .01, 95% CI = − 1.51; 1.52, *t* = .02, *p* = .99, *I*^2^ = 90.2%) and depression (*k* = 3, *g* = − .55, 95% CI = − 1.78; 0.67, *t* = − 1.94, *p* = .19, *I*^2^ = 79.9%).

**Conclusion:**

ACT could be effective in improving weight loss but more studies are needed to ascertain its effectiveness and the underlying mechanism by which the various components influence weight-related outcomes.

**Level of evidence:**

Evidence obtained from a systematic review and meta-analysis of existing empirical studies.

**Supplementary Information:**

The online version contains supplementary material available at 10.1007/s40519-023-01535-6.

## Introduction

According to the World Health Organization, the global prevalence of overweight and obesity was estimated to be more than 1.9 billion (39%) and 650 million (13%), respectively, with that of obesity nearly tripled between 1975 and 2016 [1]. This has been attributed to the global trend of increasingly unhealthy eating habits (particularly in the consumption of energy-dense, high-fat and sugary food) and sedentary lifestyles [2], leading to chronic health consequences, such as cardiovascular diseases and type 2 diabetes mellitus [3]

To improve dietary and physical activity habits, numerous interventions have been implemented by multiple stakeholders at the global, regional, and local levels. These include population-wide surveillance systems, education strategies (e.g., the Centres for Disease Control and Prevention’s Framework for Obesity prevention) [4] and individual lifestyle adaptations (e.g. using standing desks during work) [5]. However, dietary modifications  are challenging for many people with overweight and obesity as it requires a large amount of self-regulation to restrict their calorie intake [6]. This is worsened by common maladaptive eating behaviours such as emotional eating, external eating, and restraint eating, which, respectively, refer to disordered eating in response to emotions (e.g., stress and sadness) [7], external stimuli (e.g., visual and smell) [8], and the cycle of food intake restriction and indulgence [9].

Acceptance and Commitment Therapy (ACT) is a third-wave cognitive behaviour therapy that has been shown to improve both mental and physical conditions including anxiety [10], depression [11], stress [12, 13] and chronic pain [14]. At the core of ACT is the strategy to cope with averse internal thoughts, feelings and bodily sensations by exercising mindfulness and psychological flexibility [15]. Strategies to hone psychological flexibility includes acceptance, cognitive diffusion, being present, self-as- context, values and committed action [16]. In the context of weight management, ACT could improve one’s ability to cope with the negative psycho-behavioural effects of food cravings by taking these psychological experiences into perspective and committing to healthy behaviours that are coherent with the individual’s value-based goals [17, 18] This was hypothesized to improve weight management by improving the acknowledgement, acceptance and self-regulation of overeating impulses. This also prevents one from feeling negative due to a dietary lapse event (i.e., unintended eating), which could trigger an overeating episode [16].

Several systematic reviews have examined the effectiveness of ACT for weight-related behaviours. In 2020, a systematic review and network meta-analysis with adults who were overweight or obese showed that ACT has the most consistent effects on improving weight loss beyond 18 months as compared to mindfulness-based cognitive behavioural therapy (MBCT), compassion-focused therapy (CFT), and dialectical behaviour therapy (DBT) [19]. However, the effects of ACT on the various outcomes were unclear. Another meta-analysis on a general adult population showed that ACT improved physical activity in the general population but little is known about its effects on people with overweight and obesity [20]. Moreover, the variability in the types of physical activity examined across the studies may have impeded the comparability of the findings. Another systematic review with adults who were overweight or obese showed mixed findings on the effectiveness of ACT on psychological well-being (e.g., quality of life [QoL], depression, stigma), eating behaviours and weight loss [21]. This could be due to the inclusion of articles that compared the effectiveness of ACT with control group conditions that receive standard behavioural therapy (SBT) and no intervention, where the mean differences between ACT and no intervention are likely larger and hence statistically significant as compared to SBT.

To the authors’ best knowledge, there has been no meta-analysis on the effectiveness of ACT-based interventions on weight, eating behaviours, and the quality of life of those with overweight and obesity. Hence, the current study addresses this knowledge gap and provides an important review of this growing body of literature.

## Methods

The Preferred Reporting Items for Systematic Reviews and Meta-Analysis (PRISMA) guideline (Appendix 1) was used to structure this manuscript, which was registered with the International Prospective Register of Systematic Reviews (CRD42022341994). Cohen’s Kappa (*κ*) was used to assess the interrater agreements for the selection of articles and the risk of bias—*κ* = 0.00–0.20 (slight agreement); *κ* = 0.21–0.40 (fair agreement); *κ* = 0.41–0.60 (moderate agreement); *κ* = 0.61–0.80 (substantial agreement); *κ* = 0.81–1.00 (almost perfect agreement) [22].

### Search strategy

A systematic search was conducted for relevant articles published from the inception of the journal through 17 Jun 2022 across seven databases (CINAHL, EMBASE, PubMed, PsycInfo Scopus, The Cochrane Library, and Web of Science). Keywords such as acceptance and commitment therapy, ACT, body weight, body mass index, overweight and obese were stringed differently across the different databases (Appendix 2). Reference lists of the included studies were also hand-searched for relevant articles. Citations were managed using the Endnote 20 software.

### Study selection

Titles and abstracts were first screened by HSJC. Full texts of potential articles were independently assessed for inclusion eligibility by NN and CKH. Any discrepancies were resolved through consensus with a third reviewer (HSJC). Articles were included if they (1) included adults with overweight and obesity; (2) examined the effects of ACT on weight; (3) reported on at least weight loss as an outcome; (4) were randomized controlled trials; and (5) were published in English or Mandarin. Articles were excluded if they (1) included participants below the age of 18; (2) were focused on the use of ACT for weight gain; (3) examined the effectiveness of acceptance-based therapy without a component on the commitment to live a valued life.

### Data extraction

Data extraction was carried out using an excel spreadsheet template that included the following headings: authors, study design, country, sample size, sample characteristics, inclusion Body Mass Index (BMI), exclusion criteria, mean age, mean baseline BMI, percentage of male participants, theoretical underpinning, follow-up timepoints, and interventionist, if interventionists were trained in ACT, mean and standard deviation of post-interventional weight, eating behaviours and quality of life measures of each control and treatment arms. In treatment arms, the following information was extracted, including the modality of intervention (face-to-face, online, mobile; group-based, individual-based), total number of sessions, duration per session and the entire intervention, fidelity assessment and treatment adherence. The data extracted from the studies and interventions within were selected to provide a detailed view of how the studies and interventions were conducted. We separated the assessment of BMI and weight changes in mean differences instead of combining them using standardized mean differences to allow for a more meaningful interpretation for real life relevance.

### Methodological quality assessment

The Cochrane Risk of Bias (ROB) tool was used to assess the articles’ methodological quality by rating them as low, unclear, and high ROB based on six domains. These six domains consist of random sequence generation, allocation concealment, blinding of participants and personnel, blinding of outcome assessment, outcome data completeness and selective reporting. Methodological quality assessment was performed independently by CC and CKH. Discrepancies were resolved by a third reviewer (HSJC).

### Data analysis

Interventional effects estimated were pooled using random effects models by Hartung–Knapp–Sidik–Jonkman (HKSJ) to reduce the risk of obtaining false positive results, given the dearth in high-quality interventional studies on this topic [23]. Pooled effect sizes were calculated as weighted mean differences (WMD) and 95% confidence interval (CI) for BMI and weight. Standardized mean differences (SMD) corrected to Hedges’ *g* for a small sample size was calculated for binge eating, emotional eating, external eating, restraint, quality of life (QoL), depression, psychological flexibility, and stigma. Heterogeneity was assessed using the Cochrane Q statistics and quantified using the *I*^2^ statistic, where 25%, 50%, and 75% indicate a small, moderate, and large heterogeneity [24]. Sensitivity analyses were performed based on the leave-one-out approach to examine the effects of each study on the pooled effect size estimates. Subgroup analysis and the assessment for publication bias were planned for meta-analyses that included at least 10 articles but were not conducted due to the limited number (< 10) of studies included in each meta-analysis, which has a high risk of being underpowered [25]. All statistical analyses were performed using R version 4.1.3.

## Results

### Study characteristics

**Fig. 1:**
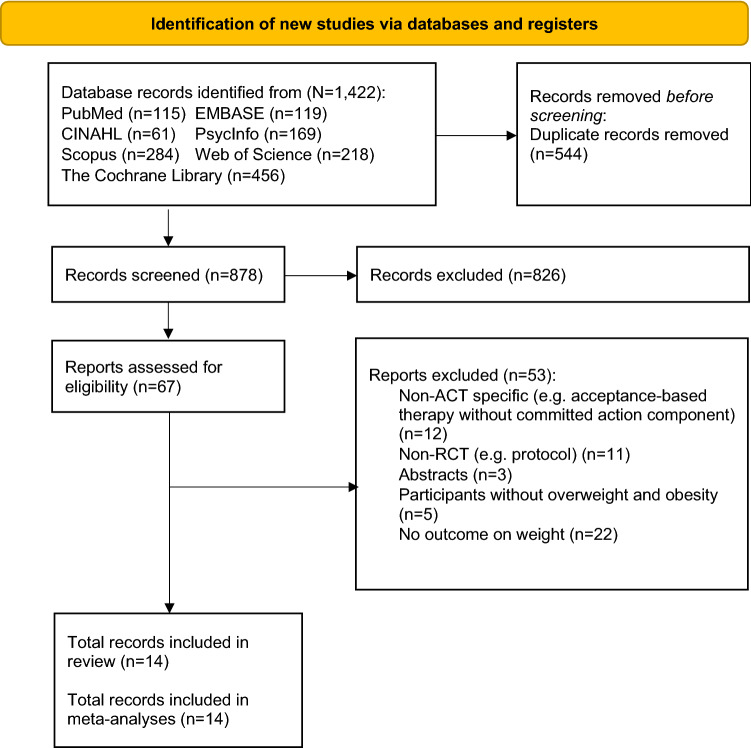
Flow diagram of the search strategy

Eleven articles were included (Fig. 1), representing 1670 participants with a mean age of 47.8 years and a mean BMI of 35.7 kg/m^2^. Most of the studies were conducted in the USA (*n* = 8, 72.7%) [26–33] except one from UK [34], Portugal [35], and Finland [36]. Three articles were conducted on solely women (*n* = 3, 27.3%) [27, 28, 33] and seven articles reported information on the sociodemographic and/or education level of the participants (*n* = 7, 63.6%) [26, 27, 31], [32, 34, 35, 36. Five articles reported the use of theoretical frameworks, namely, the cognitive behavioural model [26, 28–31] and intrinsic motivation theory [28, 29]. The attrition rate by the time of the final follow-up ranged from 0 [33] to 28.9% [30], and the follow-up timepoints ranged from [33] 2 to 36 months [30]. Five articles reported three-arm studies [26, 27, 33, 34, 36] and two articles reported different results from the same study by analyzing the data from different sub-samples [28, 29]. For three-arm studies, only intervention arms that include ACT will be included in the analysis [33, 36]. Therefore, a total of 13 studies and 48 unique effect sizes were analyzed. The interrater agreement for study selection and ROB assessment was *κ* = 0.82, *p* < 0.001 and *κ* = 0.85, *p* < 0.001, suggesting almost perfect agreement. Table 1 summarises the study characteristics of the included studies and Table 2 summarises the intervention characteristics.

**Table 1:** Study characteristics of the 11 included articles

Authors, year	No. of trial arms	Country	Sample characteristics	Sample size	Mean age	Proportion of males, %	Mean baseline BMI, kg/m^2^	^a^Attrition rate %/^b^Presence of group differences	Missing data management/Protocol registration/Funding	Report on SES/Educational level	Subject adherence, mean/%
Afari, 2019 [26]	3	USA	US veterans and self-identified as having problems with stress-related eating	88	57.3	76.1	37.2	4.5 /Yes	NS/Yes/Yes	Yes/Yes	IG: 3.5/4 CG: 3.8/4
Berman, 2022 [27]	3	USA	Women with MDD	19	51.0	0.0	42.0	21.1/NS	NS/Yes/Yes	Yes/Yes	IG: 7.1 CG: 3.3
Forman, 2013 [28]	2	USA	General overweight and obese	128	45.7	0.0	34.1	21.9/Yes	NS/Yes/Yes	No/No	IG: 21.1 CG:20.0
Forman, 2016 [29]	2	USA	General overweight and obese	190	51.6	17.9	36.9	16.0/Yes	Yes/Yes/Yes	NS/NS	IG: 21.3 CG: 20.9
Forman, 2019 [30]	2	USA	General overweight and obese	190	51.6	17.9	36.9	28.9/No	Yes/Yes/Yes	NS/NS	IG: 74.0% CG: 67.8%
Levin, 2020 [31]	2	USA	General overweight and obese	79	39.6	17.7	33.8	NS/Yes	NS/Yes/Yes	Yes/Yes	NS
Lillis, 2016 [32]	2	USA	People with high internal disinhibition	162	NS	12	37.6	0.0/No	Yes/Yes/Yes	Yes/Yes	IG:89% CG:89%
Mueller, 2022 34	3	United Kingdom	General overweight and obese	388	50.7	21.6	34.8	16.5/No	Yes/Yes/Yes	Yes/Yes	IG:32.3% CG: NS
Palmeira, 2017 [35]	2	Portugal	Women struggling with their weight	73	42.4	0.0	34.3	14.5/Yes	Yes/Yes/Yes	Yes/Yes	IG: 10.9 CG: NS
Potts, 2022 (1) [33]	3	USA	Adults high in weight self-stigma	55	38.7	18.2	37.0	0.0/No	Yes/NS/Yes	NS/NS	NS
Potts, 2022 (2) [33]	3	USA	General overweight and obese	55	38.7	18.2	37.0	0.0/No	Yes/NS/Yes	NS/NS	NS
Sairanen, 2017 [36]	3	Finland	General overweight and obese	298	49.5	15.5	31.3	12.8/No	Yes/NS/Yes	NS/Yes	NS

*BMI* body mass index; *SES* socioeconomic status; *IG* intervention group; *CG* control group; *NS* not specified

^a^Attrition rate by last outcome measurement timepoint

^b^Group differences among participants who were retained and dropped out

**Table 2:** Intervention characteristics of the 13 included studies

Authors, year	Theory	Group/Individual	Duration	Coaching sessions	Follow-up timepoint	Interventionist	Interventionist trained in ACT?	Mode of delivery	Control condition
Afari 2019 [26]	Cognitive Behavioural Model	Group	2 months	4 sessions	3 and 6 months	Full-time staff psychologist, psychology postdoctoral fellows and psychology master student	Yes	F2f group sessions	SBT
Berman 2022 [27]	NS	Group	11 weeks	11 Sessions and 9 physical sessions (before each counselling session)	3, 6, 9, 12 months	PhD psychologist	NS	F2F group meetings and physical movement sessions	Weight Watchers
Forman 2013 [28]	Intrinsic motivation theory, Cognitive Behavioural Model	Group	10 months	30 sessions	2.5, 5, 6, 10 months	Novice: Advanced doctoral students Expert: Clinical psychologists	Yes (trained in both ABT and SBT, experienced in behavioural weight-control intervention)	F2f group-based sessions	SBT
Forman 2016 [29]	Intrinsic motivation theory, Cognitive Behavioural Model	Group	12 months	25 sessions	6, 12 months	Doctoral-level clinicians	NS	F2F group sessions	SBT
Forman 2019 [30]	Cognitive Behavioural Model	Group	12 months	25 sessions	6, 12, 24, 36 months	Doctoral-level clinicians	NS (experienced in delivering behavioural weight-loss treatments)	F2F closed group sessions	SBT
Levin 2020 31	Cognitive Behavioural Model	Individual	2 months	8 sessions on canvas and 8 coaching calls	2 months	Doctoral student in clinical/counselling psychology	NS	Online (Canvas) / Coaching calls	Waitlist control
Lillis 2016 [32]	NS	Group	12 months	32 sessions	12–24 months	Mix of Ph.D. psychologists, Ph.D. exercise psychologist, and master's level nutritionist	Yes (Group leaders received training in ABT)	F2f group sessions	SBT
Mueller 2022 [34]	NS	Individual	4 months	12 sessions	4 months	Trained non-specialist	Yes (online training involved clarification of ACT Principles)	Web platform, scripted telephone call and tailored email	Waitlist control (standard advice)
Palmeira 2017 [35]	NS	Group	3.5 months	12 sessions	3.5 months (Terminus of intervention group)	Clinical psychologists and clinical psychology master student	NS (Previously trained in CBT)	F2F group sessions	Treatment as usual
Potts 2022 (1) [33]	NS	Individual	2 months	0	2 months	Advanced clinical/counselling psychology doctoral student	NS	Reading, journaling and quiz	Waitlist control
Potts, 2022 (2) [33]	NS	Individual	2 months	8 sessions	2 months	Advanced clinical/counselling psychology doctoral student	NS	Phone coaching session	Waitlist control
Sairanen 2017 (1) [36]		Group	2 months	6 sessions	9 months	NS	NS	F2F group sessions	No treatment
Sairanen 2017 (2) [36]		Group	2 months	0	9 months	NS	NS	Smartphone application	No treatment

*ABT* acceptance-based behavioral treatments; *F2F* face to face; *SBT* standard behavioural therapy; *CBT* cognitive behavioral therapy

### Methodological quality

The majority of the articles had an overall rating of having an unclear ROB (*n* = 7, 63.6%) [26, 27], [32, 33, 36, where four were rated as having a high risk of bias (*n* = 4, 36.4%) [28, 31, 34, 35]. The high ROB was attributed to the incomplete outcome data presented (*n* = 4, 36.4%) [28, 31, 34, 35], lack of blinding of participants and personnel (*n* = 2, 18.2%) [31, 34], and blinding of outcome assessment (*n* = 1, 9.1%) [34].

### ACT intervention characteristics

A summary of the intervention characteristics is shown in Table 2. Most of the 13 studies reported delivering the interventions in groups, while only four studies reported the use of individual sessions (*n* = 4, 30.8%) [31, 33, 34]. Most of the interventions were conducted face-to-face, except five studies that reported the intervention delivery through reading and journaling[33] websites [34], [31] smartphone apps [36], calls [31, 34, 33] and email information [34]. The duration and number of sessions ranged from two [26, 31, 33, 36] to 12 months [29, 30, 32], and 4 [26] to 32 sessions, respectively [32]. Most of the interventionists were clinical psychologists and psychologists in training, three studies used clinicians and laymen coaches as the interventionists [29, 30, 34] and two studies did not specify [36]. Only one study explicitly mentioned that the interventionists were being trained in learning ACT principles [34] and two mentioned training in acceptance-based therapy [28, 32]. The control group conditions were standard behavioural therapy that includes behaviour change techniques, such as cognitive restructuring and emotional self-regulation [26, 28–30, 32], treatment as usual [35], waitlist control and no treatment [31, 34, 33], [36] and the Weight Watchers program [27].

### Weight

**Fig. 2:**
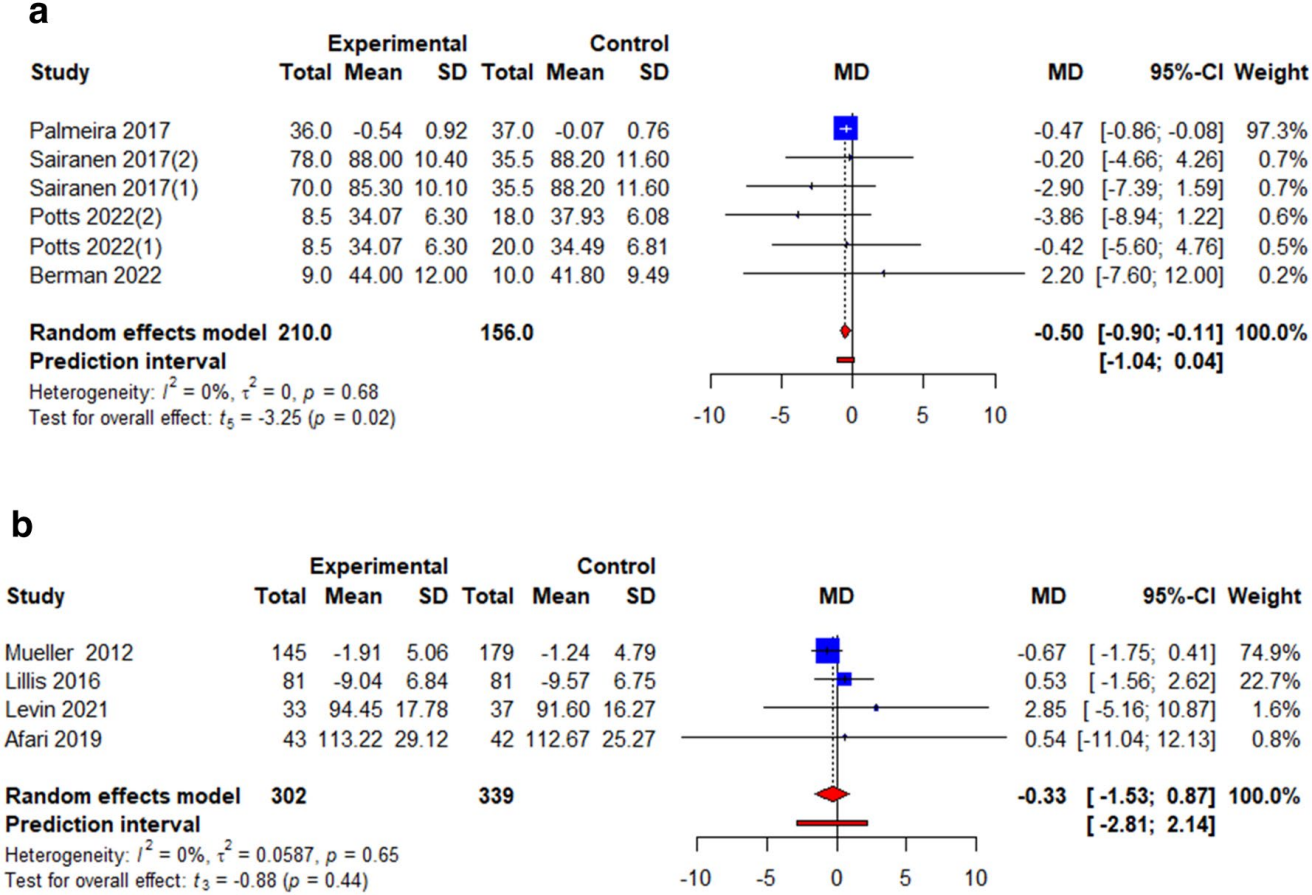
**a** Illustration of the summary statistics of the intervention and control groups in each study included in the meta-analysis on the effect of ACT on BMI. *MD* mean difference. **b** Illustration of the summary statistics of the intervention and control groups in each study included in the meta-analysis on the effect of ACT on weight, kg. *MD* mean difference

All studies reported the effects of ACT on weight loss but only those that reported weight in terms kg or kg/m^2^ were analyzed to present pooled effect estimates in terms of WMD [26, 27, 31], [32–36]. The pooled effect estimates showed significant interventional effects on BMI post-intervention (*k* = 6, WMD = − 0.50, 95% CI = − 0.90; − 0.11, *t* = − 3.25, *p* = 0.02, *I*^2^ = 0.0%) (Fig. 2a, Table 3) but not weight (*k* = 4, WMD = − 0.33 95% CI = − 1.53; 0.87, *t* = − 0.88, *p* = 0.44, *I*^2^ = 0.0%) (Fig. 2b, Table 3).

**Table 3:** Summary of meta-analyses results on each outcome at each timepoint analysed

Outcomes	*k*	WMD/SMD^a^, 95% CI	*t*	*p* value	Tau^2^	*I*^2^, %
BMI, kg/m^2^	6	− 0.50 (− 0.90; − 0.11)	− 3.25	0.02*	0.00	0.00
Weight, kg	4	− 0.33 (− 1.53; 0.87)	− 0.88	0.44	0.06	0.00
Binge Eating	4	^a^− 0.34 (− 1.31; 0.62)	− 1.13	0.34	0.27	71.1
Emotional Eating	6	^a^− 0.20 (− 0.54; 0.15)	− 1.47	0.20	0.06	45.0
External Eating	5	^a^− 0.40 (0.96; 0.16)	− 1.99	0.12	0.16	81.8
Restraint Eating	3	^a^0.22 (− 0.57; 1.01)	1.19	0.36	0.07	69.1
QoL	3	^a^0.01 (− 1.51; 1.52)	0.02	0.99	0.33	90.2
Depression	3	^a^− 0.55 (− 1.78; 0.67)	− 1.94	0.19	0.20	79.9
Psychological Flexibility	9	^a^− 0.42 (− 0.84; − 0.00)	− 2.35	0.05*	0.17	69.8
Stigma	5	^a^− 0.77 (− 1.05; − 0.50)	− 7.71	< 0.001***	0.00	0.00

*WMD* weighted mean difference

^a^*SMD* standardized mean difference adjusted with Hedges’ g; *BMI* body mass index; *p < 0.05; **p < 0.01; ***p < 0.001; *QoL* quality of life

### Eating behaviour

**Fig. 3:**
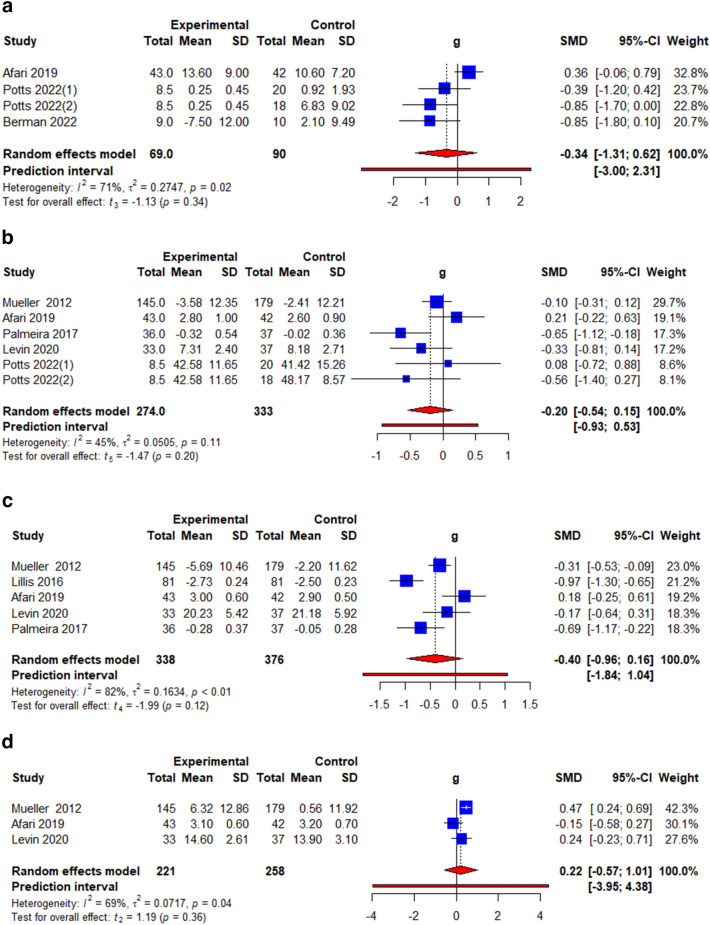
**a** Illustration of the summary statistics of the intervention and control groups in each study included in the meta-analysis on the effect of ACT on binge eating. *g* = Hedges’ *g.* **b** Illustration of the summary statistics of the intervention and control groups in each study included in the meta-analysis on the effect of ACT on emotional eating. *g* = Hedges’ *g.* **c** Illustration of the summary statistics of the intervention and control groups in each study included in the meta-analysis on the effect of ACT on external eating. *g* = Hedges’ *g.* **d** Illustration of the summary statistics of the intervention and control groups in each study included in the meta-analysis on the effect of ACT on restrained eating. *g* = Hedges’ *g*

Four eating behaviours were analyzed, namely, binge eating, emotional eating, external eating, and restrained eating. Binge eating was measured using the Binge Eating Scale (BES) [26], the Eating Disorder Diagnostic Scale (EDDS) [27] and the Eating Disorder Examination Questionnaire (EDE-Q) [33]. Emotional eating was measured using the Dutch Eating Behaviour Questionnaire (DEBQ) [26, 33], and the Three Factor Eating Questionnaire (TFEQ) [34, 35]. External eating was measured using the Dutch Eating Behaviour Questionnaire (DEBQ) [26], the Eating Inventory [32] and the Three Factor Eating Questionnaire (TFEQ) [31, 34, 35]. Restrained eating was measured using the Dutch Eating Behaviour Questionnaire (DEBQ) [26], and the Three Factor Eating Questionnaire (TFEQ) [31, 34]. The pooled effect estimates showed no significant interventional effects on binge eating (*k* = 4, *g* = − 0.34, 95% CI = − 1.31; 0.62, *t* = − 1.13, *p* = 0.34, *I*^2^ = 71.1%) (Fig. 3a, Table 3) emotional eating (*k* = 6, *g* = − 0.20, 95% CI = − 0.54; 0.15, *t* = − 1.47, *p* = 0.20, *I*^2^ = 45.0%) (Fig. 3b, Table 3), external eating (*k* = 5, *g* = − 0.40, 95% CI = 0.96; 0.16, *t* = − 1.99, *p* = 0.12, *I*^2^ = 81.8%) (Fig. 3c, Table 3) and restrained eating (*k* = 3, *g* = 0.22 95% CI = − 0.57; 1.01, *t* = 1.19, *p* = 0.36, *I*^2^ = 69.1%) (Fig. 3d, Table 3).

### Psychological outcomes

**Fig. 4:**
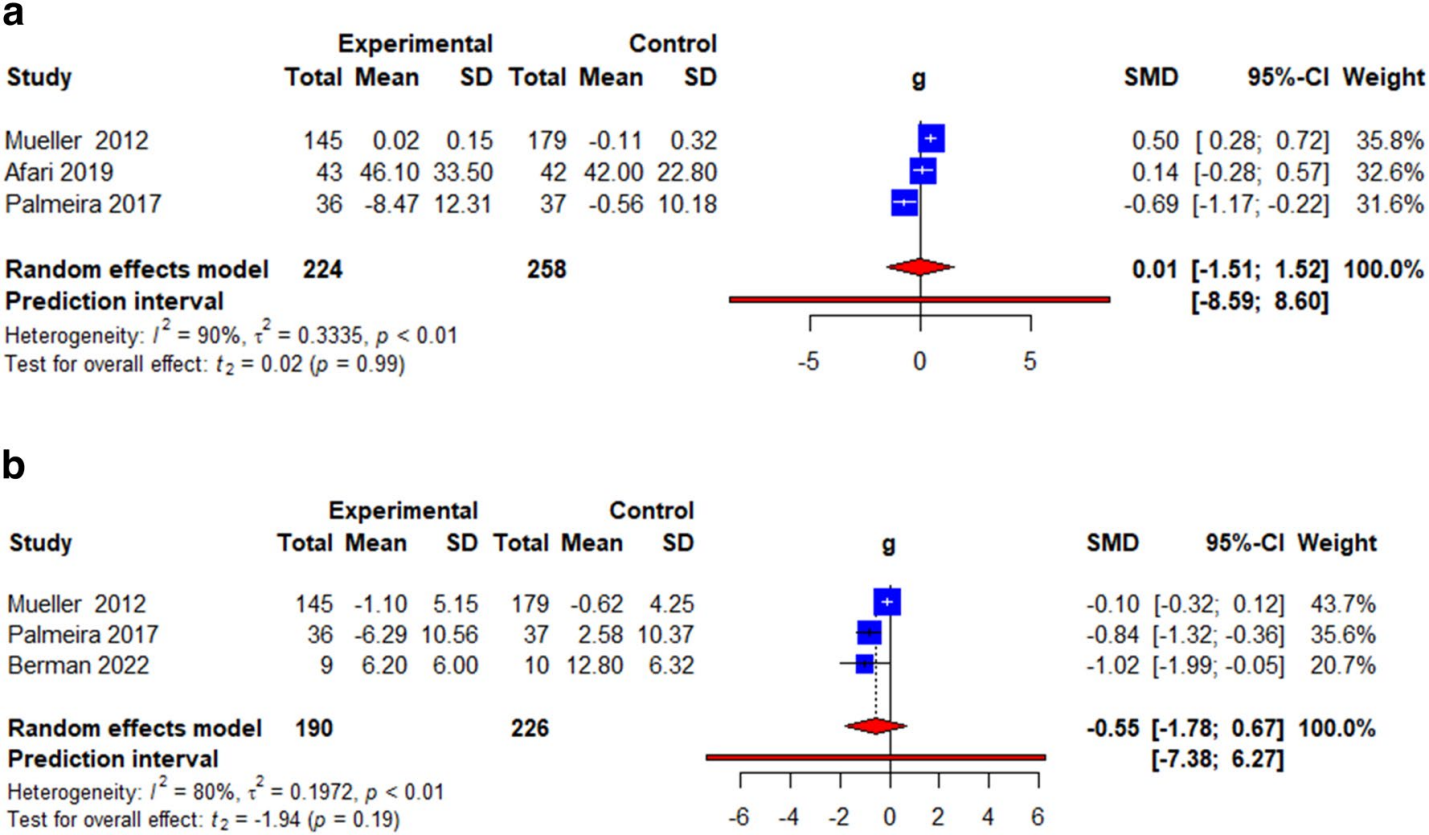
**a** Illustration of the summary statistics of the intervention and control groups in each study included in the meta-analysis on the effect of ACT on quality of life. *g* = Hedges’ *g*. **b** Illustration of the summary statistics of the intervention and control groups in each study included in the meta-analysis on the effect of ACT on depression. *g* = Hedges’ *g*

**Fig. 5:**
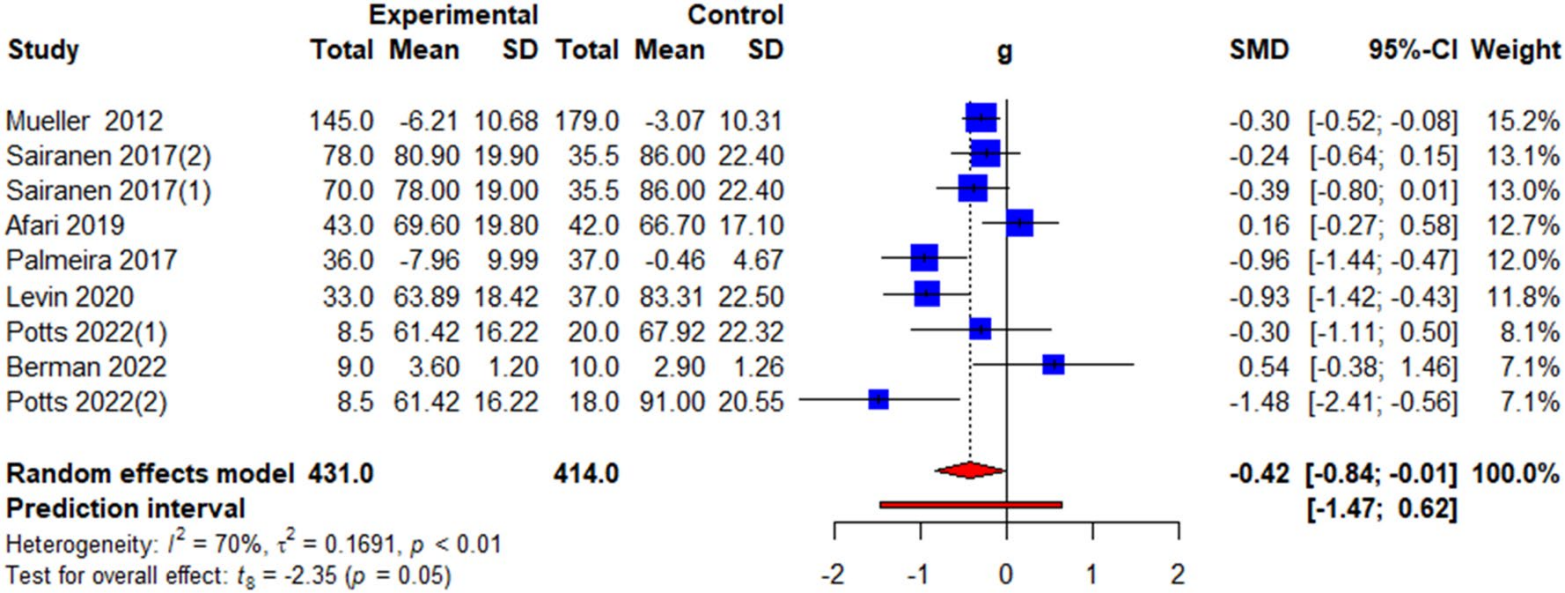
Illustration of the summary statistics of the intervention and control groups in each study included in the meta-analysis on the effect of ACT on psychological flexibility. *g* = Hedges’ *g*

**Fig. 6:**
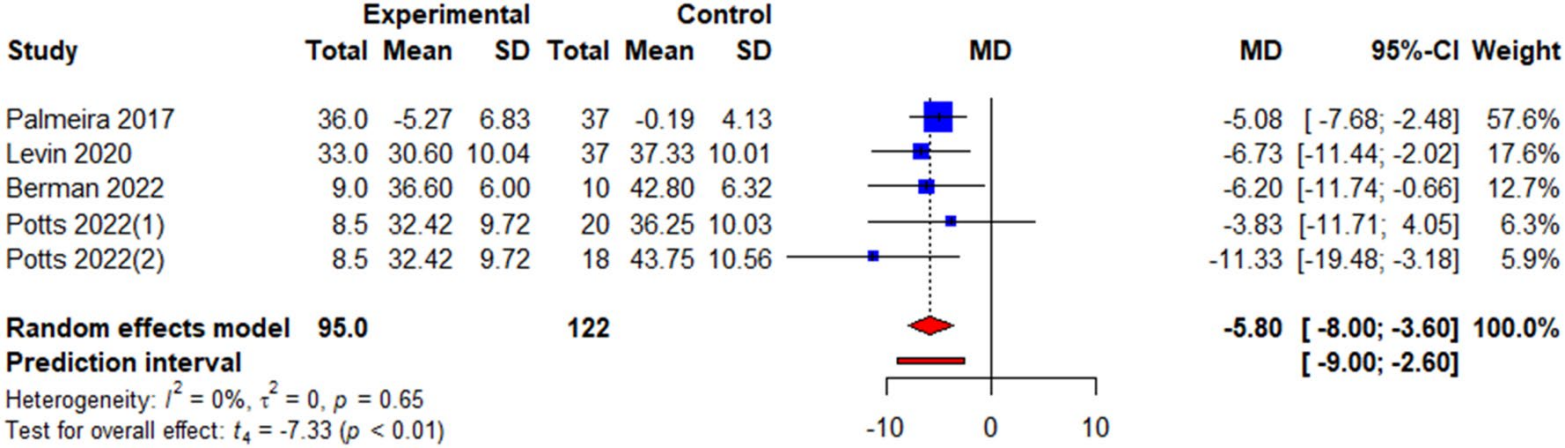
Illustration of the summary statistics of the intervention and control groups in each study included in the meta-analysis on the effect of ACT on weight stigma. *MD* mean difference

Four commonly reported psychological outcomes were analysed: quality of life, depression, psychological flexibility, and stigma. Quality of life was measured using the obesity-related well-being (ORWELL-97) questionnaire [26, 35] and ICEpop CAPability measure for Adults (ICECAP-A) [34] Depression was measured using the Hamilton Depression Rating Scale (HAM-D) [27], Patient Health Questionnaire depression scale (PHQ-8) [34] and General Health Questionnaire [35]. Psychological flexibility was measured using the Acceptance and Action Questionnaire for Weight-Related Difficulties (AAQ-W) [26, 31, 33, 36], Body Image Acceptance and Action Questionnaire (BIAAQ) [27], and AAQ-revised (AAQW-R) [34, 35]. Stigma was measured using the Weight Self-Stigma Questionnaire (WSSQ) [27, 31, 33, 35]. The pooled effects showed a non-significant change in QoL (*k* = 3, *g* = 0.01, 95% CI = − 1.51; 1.52, *t* = 0.02, *p* = 0.99, *I*^2^ = 90.2%) (Fig. 4a, Table 3) and depression (*k* = 3, *g* = − 0.55, 95% CI = − 1.78; 0.67, *t* = − 1.94, *p* = 0.19, *I*^2^ = 79.9%) (Fig. 4b, Table 3) but a significant improvement in psychological flexibility (*k* = 9, *g* = − 0.42, 95% CI = − 0.84; − 0.00, *t* = − 2.35, *p* = 0.05, *I*^2^ = 69.8%) (Fig. 5, Table 3) and stigma (*k* = 5, WMD = − 0.77, 95% CI = − 1.05; − 0.50, *t* = − 7.71, *p* < 0.001, *I*^2^ = 0.0%) (Fig. 6, Table 3).

## Discussion

To our best knowledge, this is the first systematic review that examined the effectiveness of ACT on weight, eating behaviours and psychological outcomes by pooling effect estimates through meta-analyses. Overall, our findings suggest that ACT is effective in improving certain antecedents of weight loss, such as psychological flexibility and weight-related stigma, which could eventually translate into better behavioural and emotional self-regulation of eating habits [37, 38]. However, its effect on actual eating behaviours and weight loss could be influenced by other factors including time, dietary intake, physical activity, dietary habits, and socioenvironmental circumstances that were rarely explored in the included studies [39, 40].

We found that ACT-based interventions were effective in improving weight loss in terms of BMI but not that of body mass (kg). The significant finding on BMI could be due to the overreliance on the results from one study, which was analyzed with a weight of 97.3% [35]. Therefore, we are inclined to infer that ACT has a minimal effect on body weight, especially when compared with standard behaviour treatments. This coincides with another meta-analysis that reported a non-significant effect of motivational interviewing plus cognitive behaviour therapy interventions on weight loss, suggesting the need to consider other factors that influence weight loss, such as diet and exercise [41]. The non-significant finding could also be due to the different underlying mechanisms of how ACT influences behaviour. For example, while the concept of acceptance in ACT promotes the acceptance of negative internal experiences, such as anxiety, the need for acceptance in the case of weight loss is in accepting the impending reduction in pleasure from diet and exercise modifications [17]. Future studies could consider examining this speculation and tailoring the ACT-based components for weight loss.

Only three included studies reported the delivery of ACT by personnel trained in ACT or acceptance-based therapy [28, 32, 34]. Although the students and non-specialists had received formal training for the research, they were neither professional therapists nor did they have practical ACT background apart from during their training. Although a study showed that ACT could be effectively delivered by people not trained in ACT [42], there was limited report on the direct comparability in the treatment fidelity between those with and without ACT training. On the other hand, the interventionists mentioned in the included studies could have undergone ACT training but was not reported. Future studies could consider training the interventionists in the concepts behind ACT and reporting their training status.

Acceptance- and mindfulness-based psychotherapies are theoretically grounded in East Asian philosophies [43]. However, all studies we reviewed were either from USA or Europe (UK, Portugal and Finland). Unfortunately, this is also reflective of the knowledge gap in eating behaviour-related research, where our understanding across the diverse cultures and contexts globally is currently lacking [44]. Nonetheless, there is a growing body of research on the use of ACT in non-Western cultures, such as sub-Saharan Africa [45] and China, [46, 47], though their effectiveness for intervening eating behaviours and weight management remains to be investigated.

Overall, our findings suggest modest effects of ACT on weight loss but more rigorous RCTs with larger sample sizes and more precise effect estimates are needed for more balanced meta-analyses. Having more relevant studies available would also provide adequate statistical power for subgroup analyses to estimate the effects of control group condition differences in moderating the meta-analysis results. Future studies could consider integrating concepts from ACT and behaviour change techniques that have been shown to improve weight loss to enhance the effects of current weight loss programs. Longer follow-up outcome evaluations may also produce useful information on the effects of ACT on weight loss and eating behaviour.

There are several limitations to this study. First, we only searched English databases and could have missed out on relevant articles found in databases of other languages (e.g., CNKI). Second, the reported studies had limited mention of the treatment fidelity and adherence to certain checklists for a direct comparison of intervention components and whether they can be classified as an ACT-based intervention. Despite the heterogeneity and potential issues in comparing the interventions, we focused on identifying and comparing the studies, where meaningful comparisons could be made. In addition, we identified a small subset of suitable studies for a meta-analysis of the effective on changing weight (BMI and weight), eating behaviours (binge, emotional, external and restraint eating) and psychological outcomes (quality of life, depression, psychological flexibility and stigma).

## What is already known on this subject?

- ACT is effective in improving symptoms of mental health conditions such as depression and anxiety.
- ACT could improve weight-related outcomes such as weight, body image, weight stigma and eating behaviours.

## What does this study add?

- ACT is effective in improving psychological flexibility and weight stigma in people with overweight and obesity.
- Mixed findings were found for the effectiveness of ACT on weight.
- Limited evidence supports the effectiveness of ACT on eating behaviour, quality of life and depression.

## Conclusion

Our findings suggest that the use of ACT for weight loss and eating behaviour improvements is at an early stage. The effectiveness of ACT in this application was found to be of limited effectiveness in changing weight-related outcomes and eating behaviours. The observation of wide heterogeneity in how ACT-based interventions are currently conducted indicates that future research should aim to create a standard procedure or guidance for interventions to improve their reliability and validity and to allow studies to be replicated. Finally, future research should also investigate the use of ACT in non-Western cultural contexts to address key knowledge gaps in both the ACT and general literature in weight and eating behaviour research.

## Supplementary Information

Below is the link to the electronic supplementary material.

Supplementary file1 (DOCX 27 KB)

## Data Availability

Requests for any data can be made to the corresponding author.
